# Investigation into the Production of Melanin from By-Products of Huangjiu Brewing

**DOI:** 10.3390/foods13193063

**Published:** 2024-09-26

**Authors:** Congyu Lin, Peiqi Lu, Jingqiu Ma, Tao Kan, Xiao Han, Shuangping Liu, Zhongwei Ji, Jian Mao

**Affiliations:** 1 School of Food Science and Technology, National Engineering Research Center of Cereal Fermentation and Food Biomanufacturing, Jiangnan University, Wuxi 214122, China; lincongyu@jiangnan.edu.cn (C.L.); 6230111208@stu.jiangnan.edu.cn (P.L.); hanxiao@jiangnan.edu.cn (X.H.); liushuangping668@126.com (S.L.); jizhongwei@jiangnan.edu.cn (Z.J.); 2 Jiangnan University (Shaoxing) Industrial Technology Research Institute, Shaoxing 312000, China; majingqiu1224@163.com (J.M.); kantao0610@163.com (T.K.); 3 National Engineering Research Center of Huangjiu, Zhejiang Guyuelongshan Shaoxing Wine Co., Ltd., Shaoxing 646000, China

**Keywords:** *Aureobasidium pullulans*, melanin, pullulan, huangjiu lees, resource utilization

## Abstract

Melanin is a high value bioproduct generated through the fermentation of *Aureobasidium pullulans*, playing a crucial role in various fields, including food, medicine, environmental protection, and materials science. However, its high production costs and low synthetic yields significantly limit its applications. Therefore, it is essential to identify high-yield strains, reduce production costs, and optimize fermentation strategies. In this study, a high melanin-yielding *Aureobasidium pullulans* 53LC7 was screened and identified, and the fermentation process was optimized based on melanin yield, color value, and pullulan yield. The results indicated that the melanin yield peaked at an initial pH of 6.0, temperature of 27 °C, fermentation time of 6.5 d, and inoculation quantity of 2.5%, achieving a melanin yield of 16.33 g/L. Subsequently, huangjiu lees, a byproduct of huangjiu production, was incorporated into the fermentation medium, resulting in a melanin yield of 5.91 g/L. This suggests that the *Aureobasidium pullulans* was not effectively utilizing huangjiu lees. To address this, we employed an adaptive evolution strategy, which increased the melanin yield to 8.72 g/L. The enhanced production was correlated with the expression of key genes, including *FKS*, *PKS*, and *Cmr1*. Finally, cellulase was utilized to convert the crude fibers in huangjiu lees, which were difficult to utilize, into usable substrates, while pullulanase was employed to minimize byproduct formation in the fermentation system, resulting in a melanin yield of 19.07 g/L. This study not only provides promising strains for further research but also offers valuable insights for resource production technologies.

## 1. Introduction

Huangjiu is one of the oldest known alcoholic beverages, with origins dating back to the 6th century BC [1]. Alongside wine and beer, it is recognized as one of the three ancient wines in the world [2]. Unlike other alcoholic beverages, huangjiu is distinguished by its unique brewing process and flavor characteristics [3]. It contains a variety of nutrients and beneficial compounds, such as polysaccharides and polyphenols [4], which exhibit antioxidant, hypoglycemic, and immune-enhancing properties [5]. Consequently, it is highly sought after. As the demand for huangjiu increases, the production of huangjiu lees—a byproduct of huangjiu brewing—has also risen, accounting for approximately 20–30% of the total output, which can exceed 3.5 million tons annually [6]. Due to its high content of protein, starch, and other nutrients, huangjiu lees is prone to deterioration, leading to significant environmental concerns; thus, resource utilization is imperative [7]. Currently, huangjiu lees has been utilized in applications such as animal feed and edible fungus cultivation. For instance, Yao et al. [8] investigated the use of huangjiu lees as pig feed and found that it could meet daily energy requirements; however, it was low in nutrient density and exhibited relatively poor digestibility. This indicates that the economic benefits derived from current applications are limited. Given the high-quality carbon and nitrogen sources present in huangjiu lees, there exists potential for its use as a substrate for microbial fermentation, making the synthesis of high value-added products particularly important.

Melanin is a brownish black to dark black pigment commonly found in the metabolites of various animals, plants, and microorganisms [9]. It belongs to a class of heterologous polyaromatic compounds, all of which possess complex and significant physiological properties. In recent years, the ability of fungi, bacteria, and yeast to metabolize and produce melanin with unique effects and characteristics through fermentation has become a focal point for in-depth research and practical applications. Melanin is insoluble in water, participates in redox reactions, and serves as an excellent scavenger of free radicals [10,11,12]. Additionally, it exhibits a strong capacity for light absorption and can bind with metal ions to mitigate free radical activity [13,14,15,16,17]. The distinctive properties of melanin facilitate its widespread use across various fields, including food, environmental science, cosmetics, medicine, materials science, and agriculture [18,19,20,21,22,23]. Lee et al. [24] demonstrated that the addition of melanin to the diet of high-fat diet mice improved blood lipid profiles and reduced blood glucose levels. However, the high cost, complex extraction processes, and low yield associated with obtaining melanin from plants and animals currently hinder its development and application.

*Aureobasidium pullulans*, commonly referred to as black yeast, is a polymorphic fungus characterized by a complex life history. It can exhibit various morphological forms depending on the environmental conditions and growth stages [24]. Additionally, this fungus is capable of utilizing a wide range of carbon sources, including potato residue, bagasse, corn stalks, carrot peels, and other byproducts of the food industry, to produce high-value products such as pullulan, melanin, polymalic acid, and β-glucan [25]. It is worth noting that pullulan is produced at the same time as melanin is produced [25]. Manitchotpisit et al. [26] screened 56 strains of the emerging species *Aureobasidium pullulans* from Iceland and Thailand for their ability to produce polymalic acid in a medium containing 5% (*w*/*v*) glucose, with some strains yielding high amounts of polymalic acid (9–11 g/L). Tarabasz-Szymaska et al. [27] developed *Aureobasidium pullulans* that produces non-pigmented pullulan through mutagenic irradiation, and after optimizing its fermentation conditions, the yield of pullulan reached 70 g/L. However, research on the melanin production of *Aureobasidium pullulans* remains limited. Therefore, it is crucial to select high-quality strains, enhance fermentation efficiency, and reduce production costs.

In this study, we screened and identified a melanin-producing *Aureobasidium pullulans*, 53LC7, and examined its growth and morphological characteristics. We optimized the fermentation process for melanin production to achieve the highest yield from the fermentation medium and investigated changes in color value. Subsequently, we analyzed the main components of huangjiu lees and confirmed the feasibility of using them as a fermentation substrate for melanin production. Through adaptive evolution, we developed high-yielding strains that significantly enhanced melanin yield. We also verified the expression levels of key genes and elucidated the mechanisms underlying the increased yield following adaptive evolution. Furthermore, by optimizing the fermentation strategy, we greatly improved the ability to utilize huangjiu lees as a carbon source in the fermentation substrate for efficient melanin synthesis. This approach has reduced production costs, facilitated the carbon cycle, enhanced resource efficiency, and mitigated environmental pollution.

## 2. Materials and Methods

### 2.1. Sampling and Processing

Cherry blossoms collected from Taihu Yuantouzhu in Wuxi, China (31°31′30″ N, 120°12′52″ E) were placed in a Petri dish, allowed to dry naturally, and then ground for preservation. Samples of huangjiu lees were obtained from Shaoxing huangjiu brewing enterprises in China.

### 2.2. Isolation and Culture of Strains

Screening medium: 10 g yeast extract powder, 15 g peptone, 5 g trimethylene blue, 50 g sucrose, 1 mL nalidixic acid (1 mg/L), 20 g agar, 1000 mL H_2_O, and pH 6.0. YPD medium: 20 g glucose, 10 g yeast powder, 20 g peptone, 20 g agar, and 1000 mL H_2_O. Seed Medium: 20 g sucrose, 0.6 g (NH_4_)_2_SO_4_, 2.5 g yeast powder, 0.2 g MgSO_4_, 5 g K_2_HPO_4_, 1 g NaCl, 1000 mL H_2_O, and pH 6.5. Fermentation medium: 50 g sucrose, 0.6 g (NH_4_)_2_SO_4_, 5 g yeast powder, 1 g MgSO_4_, 5 g K_2_HPO_4_, 1 g NaCl, 1000 mL H_2_O, and pH 6.5. Huangjiu lees medium: 50 g huangjiu lees powder, 1 g (NH_4_)_2_SO_4_, 1 g MgSO_4_, 5 g K_2_HPO_4_, 1 g NaCl, and 1000 mL H_2_O.

A total of 2 g of samples were accurately weighed and dissolved in 18 mL of sterile water. The solution was then mixed with aseptic glass beads for 3 h. Subsequently, 1 mL of supernatant was diluted in gradients of 10^−4^, 10^−5^, 10^−6^ and 10^−7^, which were uniformly coated onto the screening medium using 100 μL from each gradient. The plates were cultured upside down for 24 h and then incubated at 28 °C for 72 h. Concurrently, three parallel tests were established to regularly monitor the growth of strains on the plates, allowing for the timely selection of mature single colonies, which were then transferred to the YPD medium. The isolated strains were purified through multiple generations, numbered, and documented according to their growth status and colony characteristics. Strains that conformed to the typical characteristics of *Aureobasidium pullulans* were identified and cultured in seed medium for 72 h. Subsequently, 1 mL of the culture was aspirated and stored in 70% glycerol in a refrigerator at −80 °C.

### 2.3. Screening of High-Yield Melanin Strains

As mentioned in Section 2.2, the strains conforming to the basic morphology of *Aureobasidium pullulans* were cultured in seed medium for 72 h, and the seed liquid was inserted into the fermentation medium at 2% inoculum volume and cultured in a 180 rpm shock incubator at 28 °C for 120 h, and the color changes of the medium were observed. Six bottles with a black pigment and the deepest color were selected for melanin extraction and color value determination by the 2.6 method, and the optimal high-yield strain was determined.

### 2.4. Growth Curve Drawing and Morphological Characteristics Observation of Strains

Using aseptic techniques, draw 100 μL of yeast cell suspension into a 250 mL conical flask containing 50 mL of seed culture medium. Incubate this mixture at 28 °C and 180 rpm for 12 h. Subsequently, transfer 10 mL of the culture to another 250 mL conical flask containing 40 mL of seed culture medium and continue incubation at the same conditions for an additional 12 h. Samples were collected every 2 h. For sampling, take a small volume on a sterile operating table, transfer it to a blood cell counting plate, and after allowing it to stabilize, examine it under an optical microscope to count the cells. Finally, construct a growth curve with fermentation time plotted on the horizontal axis and the logarithm of the fungal count on the vertical axis. The counting formula is as follows:

$$\mathrm{fungal}\;\mathrm{count}\;(\mathrm{per}/L)=N/5÷\left(0.1/400\times 16\right)\times 10^{3}$$

*N*: The number of fungal in the five squares.

The selected strains were cultured in YPD medium, and their morphological characteristics were analyzed through plate morphology and microscopic observation.

### 2.5. Molecular Biological Identification of Strains

Yeast genomic DNA was extracted using the Thermo Fisher Scientific™ 78,870 kit (Shanghai, China), and the primers ITS1 and ITS4 (refer to Supplementary Table S1 for a list of objects) were employed for PCR (Bioer Technology QuantGene 9600, Hangzhou, China) amplification. The PCR conditions were adapted from Lin et al. [28]. The amplified products were analyzed and compared using BLAST (2.16.0) on NCBI, and a phylogenetic tree was constructed based on the sequences with high similarity.

### 2.6. Extraction and Determination of Fermentation Products

Melanin is extracted through alkaline dissolution followed by acid precipitation. Specifically:

Treatment of microbial cells: The fermentation broth was shaken and mixed thoroughly. Fifty milliliters of the fermentation broth was combined with an equal volume of a sodium hydroxide solution (1 mol/L) and mixed well. The mixture was then incubated in a water bath at 50 °C for 30 min, followed by centrifugation at 4000 rpm for 10 min. The supernatant was collected for subsequent treatment, while the precipitate comprised the microbial cells.

Acid precipitation: Hydrochloric acid solution (1 mol/L) was added to the collected supernatant to adjust the pH to between 2 and 3. The solution was thoroughly mixed and allowed to stand for 24 h to ensure complete precipitation. Following this, centrifugation was performed at 5000 rpm for 20 min, and the supernatant was discarded. The resulting precipitate was identified as crude melanin.

Purification of melanin: The crude black extract was collected and dried in a vacuum oven at 60 °C until a constant weight was achieved. It was then washed with distilled water 3 to 5 times. After drying, the sample underwent repeated cycles of alkali dissolution and acid precipitation. It was subsequently washed with a large volume of deionized water until the Cl^−^ test returned negative. Finally, the sample was vacuum dried to yield high-purity melanin.

The color value of the melanin obtained in the previous step was determined as follows. First, a 0.1 g sample of melanin was accurately weighed and dissolved in an ammonia-ammonium chloride buffer at pH 10.0, bringing the total volume to 100 mL. After oscillating for 30 min, 10 mL of the solution was transferred to a 100 mL volumetric flask, and the same ammonia-ammonium chloride buffer was added to make up to volume. Following thorough mixing, the absorbance was measured at 540 nm using an ultraviolet spectrophotometer. The resulting melanin color value was calculated using the following formula:

$$E_{1\mathrm{cm}}^{1\%}=A\times r/100W$$

*A* represents the light absorption value, *W* denotes the weight of the melanin sample, and *r* indicates the dilution ratio of the melanin sample used to determine the absorption value at 540 nm.

The determination of pullulan was conducted in accordance with the methodology outlined by Zhang et al. [29]. The residual fermentation liquid was heated to 80 °C for 15 min, followed by centrifugation at 5000 rpm for 10 min after cooling. Subsequently, twice the volume of ethanol was added to the supernatant. After standing for 12 h, the mixture was centrifuged at 12,000 rpm for 10 min, and the precipitate was dried at 80 °C until a constant weight was achieved, which represented the amount of pullulan.

### 2.7. Optimization of Fermentation Conditions of Aureobasidium pullulans

The factors selected for investigation included initial pH, temperature, fermentation time, rotational speed, and inoculation quantity. A shaking flask fermentation experiment was conducted utilizing a fermentation medium, with melanin yield, color value, and pullulan yield serving as the evaluation indices. The basic parameters are initial pH 6.0, temperature 28 °C, fermentation time 5 d, rotational speed 180 rpm, and inoculation quantity 2.0%. For further details, refer to Table 1.

Based on a single-factor analysis, we selected four factors that significantly influence the outcomes to conduct response surface experiments and optimize the process parameters. The specific design is presented in Table 2.

### 2.8. Determination of Main Components of Huangjiu Lees

The huangjiu lees sample was crushed and dried at a constant temperature of 80 °C until it reached a constant weight. The difference in weight was calculated to determine the amount of water lost. Subsequently, the sample was placed in a calcining pan within a muffle furnace and burned at 550 °C for 1 h. After combustion, the sample was cooled to room temperature in a dryer, and the total ash content was measured by weight. The crude fat content was extracted from the sample using the Soxhlet extraction method. Following this extraction, the crude protein content was determined using a Kjeldahl nitrogen analyzer. The starch content in the huangjiu lees was measured using the Total Starch Assay Kit (ZIKER181, Shenzhen Ziker Biological Technology Co., Led, Shenzhen, China), following the manufacturer’s instructions. The sample underwent a series of treatments: it was degreased with ether, neutralized after treatment with sulfuric acid, neutralized again after treatment with sodium hydroxide, and then neutralized once more after treatment with ethanol. Finally, the sample was dried at a low temperature, cooled to room temperature, and burned in a high-temperature furnace for 2 h. The difference in weight was calculated to determine the amount of crude fiber after cooling [30,31].

### 2.9. Adaptive Evolution of Aureobasidium pullulans

The adaptive evolution of *Aureobasidium pullulans* 53LC7 was conducted by gradually substituting the carbon source in the fermentation medium with 10% (*w*/*v*) huangjiu lees. After 10 successive generations of inoculation, the carbon sources in the fermentation medium were entirely replaced by huangjiu lees. Subsequently, strains from generations 1 to 10 were simultaneously introduced into the huangjiu lees medium to evaluate the effects of adaptive evolution. The optimal adaptive evolutionary strain was identified by comparing melanin yield, color value, and pullulan yield.

RT-qPCR (ViiA7, Thermo Fisher Scientific, Waltham, MA, USA) was employed to compare the transcriptional levels of *FKS*, *PKS*, and *Cmr1*, which are key genes involved in melanin synthesis in *Aureobasidium pullulans* 53LC7 during adaptive evolution. Total RNA was extracted using a kit from Sangon Biotech (B518659-0050). The EasyScript First-Strand cDNA Synthesis SuperMix (TransGen Biotech, Beijing, China) was utilized for reverse transcription to synthesize cDNA. The OLIGO 7 software was used to design the amplification primers, and the list of primers is provided in Supplementary Table S2. RT-qPCR was conducted using GoTaq qPCR Master Mix, and expression levels were evaluated using the 2^−ΔΔCT^ method.

### 2.10. Optimization of Fermentation Strategy for Huangjiu Lees

A 5 L fermentation tank was utilized for the fermentation experiment aimed at the resource utilization of huangjiu lees. The genetically stable *Aureobasidium pullulans*, obtained in Section 2.9, was inoculated into the fermentation tank containing 3 L of huangjiu lees culture medium, following high-temperature sterilization.

After enzymolysis, huangjiu lees was utilized for fermentation. Different amounts of cellulase (0.3, 0.6, 0.9, 1.2, and 1.5 g at 50 U/mg) were added to a fermenting tank containing 3 L of huangjiu lees medium. Enzymolysis was conducted at 40 °C for 30 min. The optimal enzyme concentration was determined by comparing the reduction in cellulose content following enzymolysis. Subsequently, 0.9 g of cellulase was added to the fermenter containing 3 L of huangjiu lees medium, and enzymolysis was carried out at 40 °C for varying durations of 10, 20, 30, 40, and 50 min. The optimal enzymolysis time was established by calculating the cellulose reduction after each treatment. The optimal conditions from the previous study were applied for enzymatic hydrolysis, after which the fermenter was sterilized at high temperature. Following sterilization, *Aureobasidium pullulans* was introduced for the fermentation experiment. The fermentation parameters included pH of 6.0, temperature of 27 °C, fermentation time of 6.5 d, inoculation quantity of 2.5%, ventilation rate of 2.0 vvm, and a stirring speed of 300 rpm. The yields of melanin, color value, and pullulan were compared between the two methods.

Using enzymatic hydrolysis followed by fermentation, 0.1, 0.2, 0.3, 0.4, and 0.5 g of pullulanase (1000 u/g) was added to the fermentation broth on the third day of fermentation. By comparing the yield and color value of melanin after 10 d of fermentation, the optimal enzyme addition amount can be determined. Additionally, 0.3 g of pullulanase was added to a fermentation tank containing 3 L of culture medium derived from the cellulase hydrolysis of huangjiu lees, and the yield and color value of melanin were measured over a fermentation period of 3 to 10 d to establish the best fermentation time.

### 2.11. Statistical Analysis

Data were averaged across three replicates and plotted using Origin Pro 2022. Statistical analyses were conducted using SPSS version 19.0. Duncan’s multiple range test was employed to compare the means of the data. *p* < 0.05 was considered statistically significant.

## 3. Results and Discussion

### 3.1. Identification of Melanin-Producing Strains

Fermentation experiments were conducted using ten selected strains that exhibited the characteristic morphology of *Aureobasidium pullulans*. Among these, two bottles of fermentation liquid were light yellow, one bottle was dark yellow, one bottle was light green, and the remaining six bottles were either black or dark green. The melanin extracted from the six bottles of fermentation liquid was analyzed and compared, as presented in Table 3. The results indicate that the melanin yield from strain 53LC7 was significantly higher than that of the other strains, and its color value was also the highest among the six strains. These findings suggest that melanin production and color value can be preliminarily inferred from the color of the fermentation solution, with a positive correlation observed between the two. Consequently, it was justifiable to exclude the four strains with light yellow, dark yellow, and light green fermentation solutions and to focus on the remaining six bottles.

### 3.2. Plotting of the Growth Curve of Strain 53LC7

**Figure 1:**
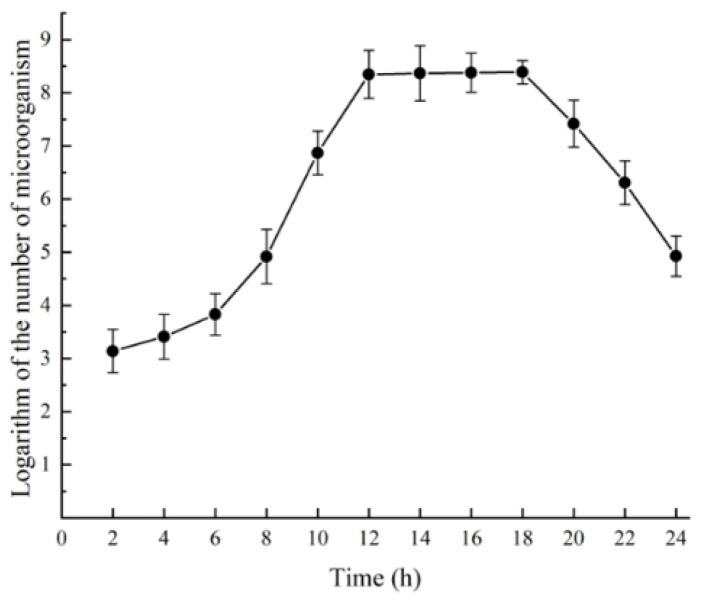
Growth curve of strain 53LC7.

Previous studies have partially investigated the growth characteristics of *Aureobasidium pullulans*. Therefore, this experiment allows for comparison with other studies to determine whether the selected strains align with the growth patterns of *Aureobasidium pullulans*. As shown in Figure 1, the number of fungal cells consistently increased within 18 h post-transfer. Specifically, from 0 to 6 h, the strain was clearly in the lag phase of growth; from 6 to 12 h, it entered the logarithmic growth phase; and during the 12 to 18-h interval, the increase in cell number was not significant, indicating that the strain was in the stationary phase. After 18 h, it entered the decline phase. This growth curve is consistent with the fundamental growth patterns of *Aureobasidium pullulans* as reported by Zhang et al. [32]. Therefore, for subsequent fermentation experiments, strains that are 6 to 12 h old should be selected for inoculation.

### 3.3. Morphological Characteristics of Strains

**Figure 2:**
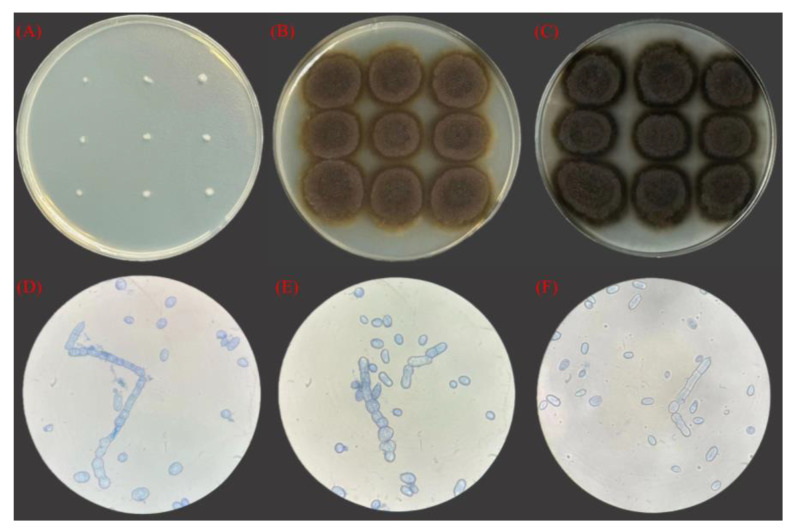
Strain morphology. (**A**–**C**) Morphology of strain 53LC7 on the 1st, 3rd and 6th day. (**D**–**F**) Morphology of strain 53LC7 on days 1, 3, and 6 under a 40 objectives optical microscope.

The strain was inoculated onto a YPD plate, as illustrated in Figure 2A, where it exhibited robust growth. On the first day, the strain appeared white, smooth, and shiny, with a small colony diameter and a typical yeast-like cell shape, resembling other yeast colonies. Over time, the morphology of the strain on the plate medium underwent notable changes. As depicted in Figure 2B, the color transitioned from white to dark green by the third day, the colony diameter significantly increased, the surface exhibited an oily luster and slight bulging, and the colony’s surrounding area radiated outward into the medium. By the sixth day, as shown in Figure 2C, further changes were observed: the colony diameter continued to expand, reaching the edge of the Petri dish, the color shifted from dark green to black, and the colony surface collapsed. The luster diminished, the center of the colony became moist, and a distinct boundary emerged between the colonies.

By observing the state of the strain on days 1, 3, and 6 under a 40 objectives optical microscope, as illustrated in Figure 2D–F, we clearly observed the morphology and developmental process of the strain, which consisted of short pedospores and budding spores. In the early stages of development, spores gradually emerge from the short stalk and proliferate. As the strain matures, spores develop independently from the short stalk, with some grouping together while others remain isolated. Notably, we observed a common phenomenon in which, as the species ages, the color of the spores transitions from an initial dark hue to a colorless and transparent state, correlating with the maturation of melanin. This observation indicates that melanin is synthesized within the cell and subsequently secreted outside through transmembrane transport.

### 3.4. Molecular Biological Identification and Phylogenetic Tree Construction

**Figure 3:**
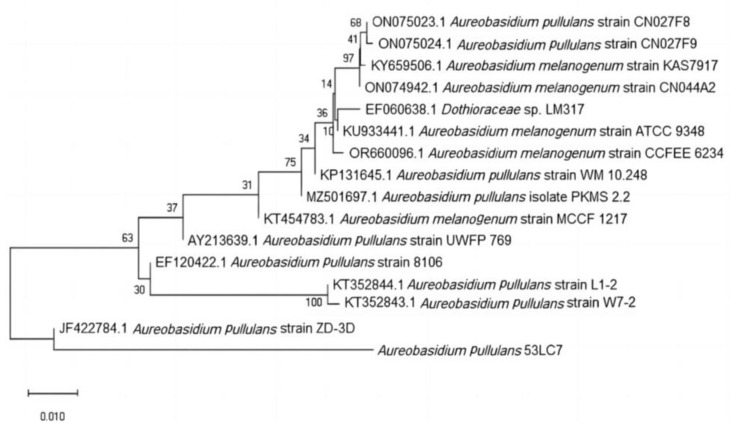
Phylogenetic tree.

Molecular biological identification of the strain was conducted through DNA extraction using a kit, followed by polymerase chain reaction (PCR) employing primers ITS1 and ITS4. The gel electrophoresis of the PCR products revealed fragment sizes ranging from 500 to 750 bp, which corresponded with the expected size of fungal ITS fragments. Subsequent sequencing yielded the ITS sequence, and a phylogenetic evolutionary tree was constructed, as illustrated in Figure 3. The results indicate that strain 53LC7 and *Aureobasidium pullulans* strain ZD-3D are located on the same branch node, forming a stable and independent branch. Similar nodes, such as *Aureobasidium pullulans* strain L1–2, *Aureobasidium pullulans* strain W7-2, and *Aureobasidium pullulans* strain 8106, also belong to the genus *Aureobasidium*. Consequently, strain 53LC7 is classified within the genus *Aureobasidium*.

### 3.5. Optimization of Fermentation Conditions for Aureobasidium pullulans 53LC7

To assess the capability of this strain to produce melanin, it is essential to optimize the fermentation conditions to maximize productivity.

#### 3.5.1. Effect of Fermentation Conditions

**Figure 4:**
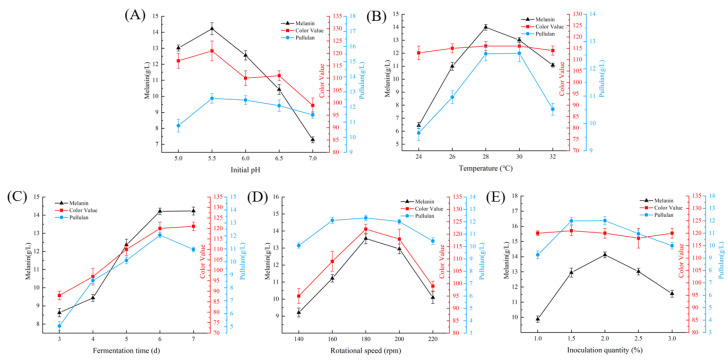
Single-factor plot. (**A**) Effect of initial pH on melanin yield, color value and pullulan yield. (**B**) The effect of temperature on melanin yield, color value and pullulan production. (**C**) The effect of fermentation time on melanin yield, color value and pullulan yield. (**D**) The effect of rotational speed on melanin yield, color value and pullulan yield. (**E**) Effects of inoculation quantity on melanin yield, color value and pullulan yield.

As illustrated in Figure 4, we investigated the relationship among initial pH, temperature, fermentation time, rotational speed, inoculation quantity, and the yields of melanin, color value, and pullulan. Figure 4A reveals that within the pH range of 5.0 to 5.5, melanin production, color value, and pullulan production exhibit an upward trend. At a pH of 5.5, these three parameters reach their maximum values; however, with further increases in pH, the production of melanin, color value, and pullulan begins to decline. These results indicate that pH significantly influences fermentation, with acidic conditions being more conducive to the accumulation of 53LC7 products. Nevertheless, as pH increases, both melanin and color value show a marked decrease, while the impact on pullulan synthesis remains relatively minor. This suggests that the effect of pH on melanin metabolism is significantly greater than its effect on pullulan synthesis.

As illustrated in Figure 4B, temperature significantly influences the synthesis of melanin and pullulan. When the temperature ranges from 24 °C to 28 °C, the yield of melanin, color value, and pullulan exhibit an increasing trend. At 28 °C, the production of all of them reach their peaks; however, as the temperature continues to rise, the production of melanin declines significantly. In contrast, both the color value and pullulan production remain relatively stable between 28 °C and 30 °C before subsequently decreasing. These findings indicate that temperature plays a crucial role in fermentation, which is essential for the synthesis of melanin and pullulan, although the color value remains largely unaffected by temperature variations.

As illustrated in Figure 4C, fermentation time significantly influences melanin yield, color value, and pullulan yield. During the fermentation period of 3 to 6 d, melanin yield, color value, and pullulan yield exhibited an upward trend. On the sixth day, the pullulan yield peaked; however, with further increases in fermentation time, the pullulan yield began to decline. Notably, after 6 d, there were no significant changes in melanin production or color value. This suggests that after a certain fermentation duration, melanin production reaches a relative equilibrium, while pullulan yield declines, potentially due to the breakdown of glycosidic bonds and the depletion of substrates necessary for cellular synthesis.

As illustrated in Figure 4D, rotational speed significantly influences the fermentation process. At rotational speeds ranging from 140 to 180 rpm, melanin yield, color value, and pullulan yield exhibit a notable increasing trend. At 180 rpm, the yields of melanin, color value, and pullulan reach their maximum levels; however, yields decline with further increases in rotational speed. This observation is based on flask experiments. It is important to note that the shear forces generated by excessively high speeds can adversely affect cell growth. Therefore, the judicious selection of fermentation equipment and the careful setting of fermentation parameters become critical in subsequent experiments.

As illustrated in Figure 4E, the inoculation quantity significantly influenced the yield of both melanin and pullulan. When the inoculation quantity ranged from 1.0% to 2.0%, yields of both substances increased. However, beyond this range, further increases in inoculation quantity led to a decline in the yields of melanin and pullulan. In contrast, the color value remained relatively unaffected by the inoculation quantity, exhibiting no significant trend. These findings indicate that within a certain range, an appropriate increase in inoculation quantity enhances the fermentation process and is positively correlated with product accumulation. Conversely, exceeding a specific threshold of inoculation quantity may divert the fermentation substrate towards the growth of strains, thereby hindering the accumulation of target products.

#### 3.5.2. Optimization of Melanin Fermentation Process by Response Surface Method

Using Design-Expert 12.0 software, we developed a binary multiple regression model to analyze the relationship between melanin yield and the factors of initial pH, temperature, fermentation time, and inoculation quantity. The resulting model for melanin yield (g/L) is expressed as follows:

Melanin yield (g/L) = 15.85 + 0.97A + 0.79B + 0.70C + 0.65D − 0.72AB + 1.22AD − 0.69BD − 2.54A^2^ − 1.21B^2^ − 2.66C^2^

The analysis revealed that the model is statistically significant, with *p* < 0.0001. In contrast, the misfit *p* value was 0.18 > 0.05, suggesting that it is not significant. Therefore, the model demonstrates a good fit and can be effectively utilized to optimize melanin yield within the specified range. The relative influence of each factor on melanin yield is ranked as follows: initial pH > temperature > fermentation time > inoculation quantity.

The response surface analysis indicated that the four selected factors significantly influenced melanin production. According to predictions generated by Design-Expert 12.0 software, the optimal conditions for achieving the highest melanin yield of 16.16 g/L occurred at an initial pH of 6.0, temperature of 27.4 °C, fermentation time of 6.3 d, and inoculation quantity of 2.5%. To validate these predictions, three sets of parallel experiments were conducted. Taking practical considerations into account, the fermentation parameters were adjusted to an initial pH of 6.0, temperature of 27 °C, fermentation time of 6.5 d, and inoculation quantity of 2.5%. The actual yield obtained was 16.33 ± 0.33 g/L, which fell within the error margin of the predicted value. These results indicate that the established regression equation accurately reflects the influence of the four factors on the melanin production fermentation process, thereby confirming the feasibility of the proposed process conditions.

### 3.6. The Main Ingredient of Huangjiu Lees

**Figure 5:**
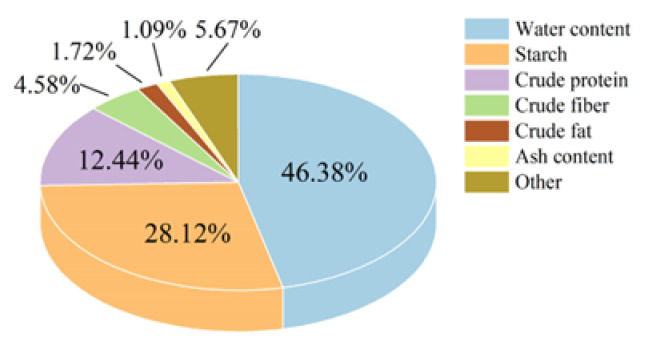
Composition of huangjiu lees.

Various types of huangjiu exist, each characterized by distinct raw materials, Qu, and starter cultures. These variations contribute to differences in the composition of huangjiu lees. Consequently, conducting relevant analyses is crucial to ensure the accuracy and rigor of this experiment. The primary components of the huangjiu lees utilized in this study are illustrated in Figure 5. The moisture content of huangjiu lees is 46.38%, which constitutes approximately half of the total composition. This finding aligns with the research conducted by Zhu et al. [33], suggesting the presence of numerous components beyond water, many of which possess significant utilization potential. For instance, huangjiu lees contain 28.12% starch, which serves as a high-quality carbon source for microbial growth. Additionally, crude protein, accounting for 12.44%, functions as an essential nitrogen source for microbial metabolism. Crude fiber can also be enzymatically hydrolyzed into substrates for microbial fermentation. Given these results, it is a considerable waste of resources to treat huangjiu lees as mere low-value waste. Based on the aforementioned results, regarding huangjiu lees as mere waste represents a significant misallocation of resources. Therefore, it warrants extensive research and high-value utilization.

### 3.7. Adaptive Evolution

The fermentation experiments were conducted using the optimal fermentation medium, which yielded the most favorable fermentation parameters and target yield, thereby providing valuable insights for the fermentation of huangjiu lees. However, as indicated in Section 3.6, the primary components of huangjiu lees reveal that, in comparison to the fermentation medium, the composition of huangjiu lees is relatively deficient; specifically, the crude fiber and other constituents cannot be utilized directly. Furthermore, the yield of melanin in the huangjiu lees fermentation medium exhibited significant differences when compared to that in the fermentation medium outlined in Section 3.5. Therefore, conducting adaptive evolution is crucial for optimizing the fermentation substrate to enhance the efficient synthesis of melanin.

#### 3.7.1. Comparison of Adaptive Evolutionary Strains

**Figure 6:**
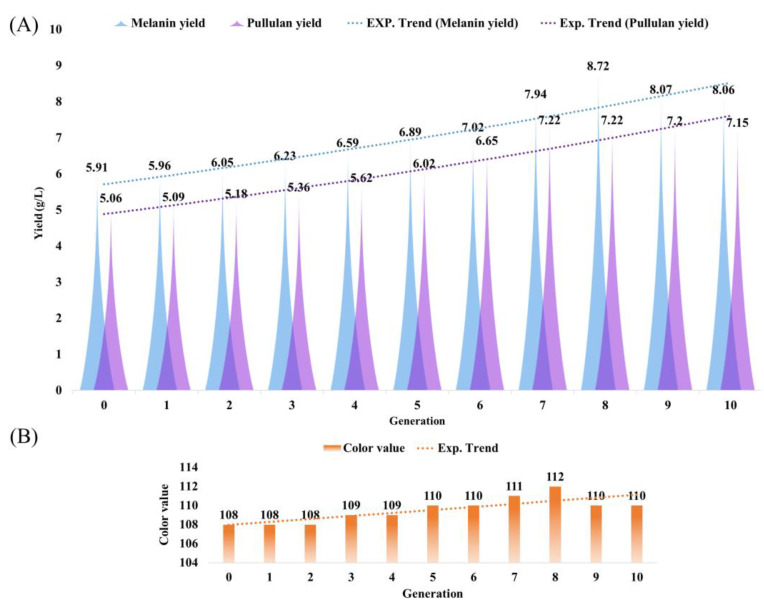
Yield difference map before and after adaptive evolution. (**A**) Maps of differences in melanin and pullulan production before and after adaptive evolution. (**B**) Color value difference map before and after adaptive evolution.

With the advancement of adaptive evolution, the ability of *Aureobasidium pullulans* 53LC7 to utilize huangjiu lees as a medium progressively improved. As presented in Supplementary Table S3, the initial melanin yield was only 5.91 g/L. However, with an increase in the number of evolutionary generations, the highest melanin yield reached 8.72 g/L in the 8th generation, demonstrating a significant effect. Concurrently, pullulan yield rose from 5.06 g/L to 7.22 g/L, which also exhibited a significant effect. Notably, the color value did not show significant changes throughout the adaptive evolution process. These results indicate that adaptive evolution significantly enhances melanin and pullulan production, whereas the increase in color value does not depend on adaptive evolution. As illustrated in Figure 6A,B, the yield differences among various generations are evident. This phenomenon may be attributed to the influence of adaptive evolution on the expression of key genes involved in the synthesis of melanin and pullulan, whereas the color value is not regulated by these genes.

#### 3.7.2. Comparison of Key Gene Expression

As previously mentioned, the *FKS*, *PKS*, and *Cmr1* genes are crucial for melanin synthesis in *Aureobasidium pullulans* 53LC7, and their expression is closely linked to melanin production. The *PKS* gene primarily functions in the synthesis of secondary metabolites, which are essential for fungal growth, development, and environmental adaptation. *Cmr1* serves as a significant transcriptional activator that regulates the melanin synthesis pathway in fungi. Similarly, *FKS* functions as a transcriptional activator that regulates the pullulan synthesis pathway in fungi.

**Figure 7:**
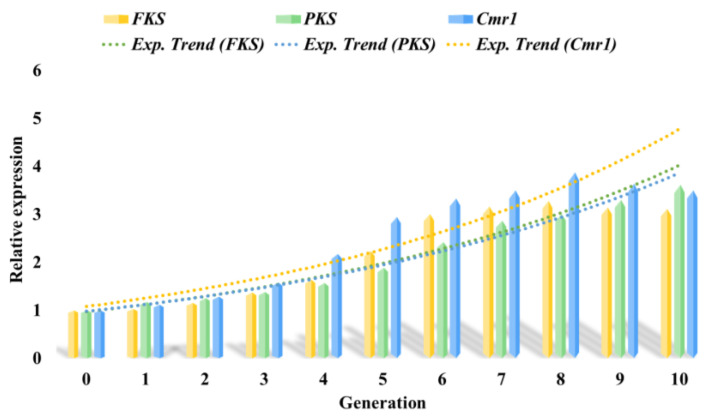
Verification of key genes.

As illustrated in Figure 7, the expression levels of *FKS*, *PKS*, and *Cmr1* varied with the incremental addition of huangjiu lees to the fermentation substrate. Analysis of the evolutionary strains from 1st to 10th generation reveals that the expressions of *FKS*, *PKS*, and *Cmr1* improved to varying extents. Notably, in the 8th generation, the expression levels of the *FKS* and *Cmr1* genes reached their peak, indicating efficient synthesis of melanin and pullulan at this stage. Conversely, the expression levels of the *PKS* gene were highest in the 10th generation, which aligns with its gene function. The growth and development of microorganisms, as well as their adaptation to environmental changes, are significantly influenced by *PKS* genes. This suggests that *Aureobasidium pullulans* 53LC7 can effectively regulate gene expression in response to environmental variations. Consequently, adaptive evolution emerges as a viable strategy for enhancing metabolite yields. Furthermore, the study elucidates the yield differences presented in Supplemental Table S3 from the perspective of gene expression.

### 3.8. Optimization of Fermentation Strategy for Production of Melanin in Huangjiu Lees

The yield of melanin produced by the fermentation of huangjiu lees with the adaptive evolution of *Aureobasidium pullulans* 53LC7 has been significantly improved, but there are still some other products that affect the metabolic process of melanin, such as pullulan, etc. At the same time, cellulose in huangjiu lees is not efficiently utilized. If these problems are solved, the yield of melanin will be improved. It is also more in line with the principle of making the best use of everything, so we have studied fermentation strategies in order to obtain higher melanin production.

#### 3.8.1. Study on Fermentation of Huangjiu Lees Treated with Cellulase

**Figure 8:**
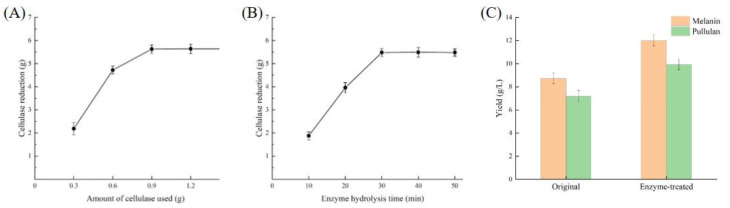
Study on treatment conditions of cellulase and results. (**A**) Effect of cellulase usage on cellulose. (**B**) Effect of enzymatic hydrolysis time on cellulose. (**C**) Comparison of fermentation yield between original medium and enzyme-treated medium.

In this experiment, a medium composed of 3 L of huangjiu lees was utilized for fermentation. According to the medium formulation calculations, the system contained 150 g of huangjiu lees powder, and 6.82 g of cellulose was determined using the 2.8 method. As illustrated in Figure 8A, the amount of cellulose gradually decreased with increasing cellulase usage. When the cellulase dosage reached 0.9 g, the cellulose content decreased to 5.63 g, after which further additions of cellulase did not result in significant changes. From an economic perspective, incorporating 0.9 g of cellulase into every 3 L of huangjiu lees medium proved to be the optimal choice. As shown in Figure 8B, cellulose levels decreased rapidly during the first 30 min, reaching 5.48 g at that time. Beyond this period, no significant changes in cellulose concentration were observed. Therefore, a treatment duration of 30 min was selected for subsequent experiments.

Fermentation parameters in 2.10 were used for fermentation verification, and the yield of melanin and pullulan produced by raw huangjiu lees medium and enzyme-treated huangjiu lees medium were investigated, respectively. The results were shown in Figure 8C. The amount of melanin produced by the fermentation of raw huangjiu lees medium was 8.74 g/L. The amount of melanin produced by the fermentation medium of huangjiu lees after enzyme treatment was 12.01 g/L, and the effect was significant. At the same time, the yield of pullulan also increased significantly, indicating that cellulase treatment played an important role in this experiment, and the treated huangjiu lees medium was more suitable for resource utilization. We also measured and studied the color value, and the results showed that the treatment did not cause significant difference to the color value.

#### 3.8.2. Investigation of Pullulanase-Assisted Treatment for Increased Melanin Production

The aforementioned studies demonstrated that fermentation produced melanin (12.01 g/L), whereas pullulan (9.92 g/L) was also produced. The synthesis of pullulan affects the production of melanin, as it competes for carbon sources in the medium and complicates subsequent separation and purification processes. Consequently, it is crucial to minimize pullulan synthesis and convert it into a reusable carbon source. Pullulan is a linear macromolecular polysaccharide formed by the polymerization of maltose triosaccharide repeat units through α-1,6-glucosidic bonds. Pullulanase can enzymatically hydrolyze pullulan into maltose triosaccharide, facilitating its reuse by *Aureobasidium pullulans* [34].

**Figure 9:**
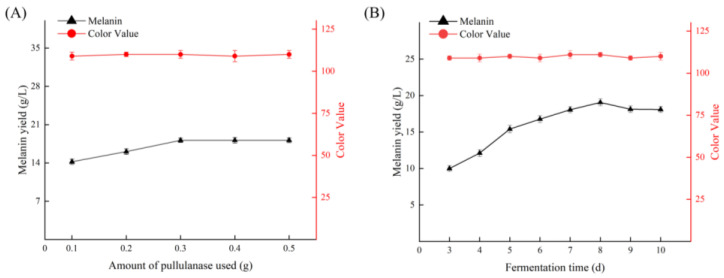
Study on treatment conditions of pullulanase. (**A**) Effect of the amount of pullulanase on melanin yield. (**B**) Effect of fermentation time on melanin yield.

As illustrated in Figure 9A, the yield of melanin increased with the addition of pullulanase. Specifically, when the amount of pullulanase reached 0.3 g, the melanin yield peaked at 18.13 g, demonstrating a significant difference compared to the control group without pullulanase. Beyond this point, further increases in enzyme concentration did not yield a significant difference in melanin production. Concurrently, we assessed the color value; the addition of pullulanase did not result in any significant changes in color value, indicating that pullulanase does not affect the color value of melanin. Figure 9B shows that melanin production exhibited a significant upward trend throughout the fermentation process, while the color value remained relatively stable. On the 8th day, melanin production reached its maximum value of 19.07 g/L before subsequently declining. Therefore, we incorporated 0.3 g of pullulanase into the 3 L huangjiu lees culture medium on the 3rd day of fermentation, which lasted for a total of 8 d. This procedure represents the optimal process. This method aims to reprocess unnecessary byproducts generated during the recycling process into substrates for production, thereby achieving complete utilization and offering novel insights for recycling production technology.

As the functional properties of melanin become increasingly understood, its potential applications continue to expand, prompting an upsurge in related research. However, high production costs and low synthesis efficiency significantly hinder the development and application of melanin. Therefore, it is crucial to identify high-yield melanin-producing strains and explore cost-effective fermentation substrates. Several studies have indicated that *Aureobasidium pullulans*, a well-known melanin-producing microorganism, is commonly referred to as black yeast [35]. In this study, we screened a melanin-producing strain, designated as 53LC7, from flowers. The strain was identified based on its morphological characteristics and molecular biological methods, and its growth conditions were investigated. The strain exhibited the highest activity in 6 to 12 h of growth, making it suitable for fermentation experiments. Notably, as illustrated in Figure 2, there were significant morphological differences among the strains at various time points on the medium, which aligns with the amorphous fungal characteristics of *Aureobasidium pullulans*. Subsequently, we optimized the fermentation culture process. Figure 4 demonstrates the relationships among initial pH, temperature, fermentation time, rotational speed, inoculation quantity, melanin yield, color value, and pullulan yield. A response surface methodology was employed for process optimization. The optimal conditions were determined to be an initial pH of 6.0, temperature of 27 °C, fermentation time of 6.5 d, and an inoculation amount of 2.5%. Under these conditions, the yield of melanin was 16.33 g/L, the color value was 110, and the pullulan yield was 11.85 g/L. These findings indicate that variations in fermentation processes significantly affect melanin yield, corroborating current research on the influence of pH and temperature on melanin production. We have conducted a more comprehensive optimization of this process, thereby establishing a foundation for the subsequent amplification experiment.

As a byproduct of huangjiu production, huangjiu lees are rich in protein and starch; however, their utilization has been limited, leading to a significant waste of resources. In this study, industrial huangjiu lees were sourced from Shaoxing, the largest huangjiu-producing region in China, and their primary components were analyzed. As illustrated in Figure 5, the huangjiu lees contained 28.12% starch, which can be considered a high-quality carbon source for microbial growth. Crude protein, comprising 12.44% of the composition, serves as an essential nitrogen source for microbial metabolism. Additionally, crude fiber can be converted into a substrate for microbial fermentation through various technical methods, highlighting the feasibility and significance of our research. Preliminary experiments indicated that *Aureobasidium pullulans* was unable to efficiently utilize the huangjiu lees medium for melanin production, necessitating further investigation to optimize its use. Through the process of adaptive evolution, we examined melanin yield, color value, and pullulan yield. As depicted in Figure 6, the impact of adaptive evolution was notable, with a 47.55% increase in melanin yield; however, this was accompanied by a simultaneous rise in pullulan yield, which was not our intended outcome. Concurrently, we analyzed the expression of key genes involved in the synthesis process. The experimental results presented in Figure 7 corroborated the outcomes of adaptive evolution, revealing changes in the *FKS*, *PKS*, and *Cmr1* genes that aligned with their respective functions, consistent with the findings of Zhang et al. [29].

As a byproduct of fermentation, pullulan is being extensively studied, whereas research on melanin is relatively limited. In fermentation systems, the production of pullulan competes with the generation of melanin for fermentation substrates, which can also affect the extraction and purification of melanin. Therefore, developing a new fermentation model to reduce the production of pullulan and convert it into reusable substrates has emerged as a new research direction. This study focuses on optimizing fermentation strategies for the huangjiu lees medium, which contains substances like cellulose that are not fully utilized by microorganisms, along with the characteristics of concomitant pullulan production. By employing a synergistic approach using cellulase and pullulanase, the study maximizes the utilization of fermentation substrates, minimizes the generation of byproducts in the fermentation system, and significantly enhances the yield of melanin. This research has important implications for industrial production and offers new strategies for the targeted high-value utilization of byproducts.

## 4. Conclusions

In this study, we first screened and identified a strain of melanin-producing yeast, *Aureobasidium pullulans* 53LC7, and investigated its growth and fermentation processes. Subsequently, we conducted adaptive evolution to enhance the utilization of huangjiu lees as a byproduct for melanin production. The variations in yield were validated through changes in the expression of key genes. Additionally, we proposed an efficient fermentation production strategy that maximizes substrate utilization and recycles byproducts, resulting in a significant increase in melanin yield from 5.91 g/L to 19.07 g/L. The methodologies developed in this study can serve as a reference for other strains aiming to synthesize high-value products from industrial production byproducts.

## Figures and Tables

**Table 1:** Parameter of fermentation conditions.

Single Factor	Variable
Initial pH	5.0, 5.5, 6.0, 6.5, 7.0
Temperature (°C)	24, 26, 28, 30, 32
Fermentation time (d)	3, 4, 5, 6, 7
Rotational speed (rpm)	140, 160, 180, 200, 220
Inoculation quantity (%)	1.0, 1.5, 2.0, 2.5, 3.0

**Table 2:** Response surface experimental factor table.

Horizontal	A: Initial pH	B: Temperature (°C)	C: Fermentation Time (d)	D: Inoculation Quantity (%)
−1	5.0	26	5	1.5
0	5.5	28	6	2.0
1	6.0	30	7	2.5

**Table 3:** Strain screening table.

Strain	Color	Melanin Yield (g/L)	Color Value
53LC2	black	15.99 ± 1.02	97
53LC5	black	15.31 ± 0.99	92
53LC7	black	17.88 ± 1.22	118
53LC8	dark green	8.09 ± 0.92	58
53LCX	black	14.89 ± 1.34	90
53LCY	black	16.39 ± 0.26	99

## Data Availability

The original contributions presented in the study are included in the article/Supplementary Material, further inquiries can be directed to the corresponding author.

## Supplementary Materials

The following supporting information can be downloaded at: https://www.mdpi.com/article/10.3390/foods13193063/s1, Table S1: ITS universal primer sequence; Table S2: Primers used in this study; Table S3: Comparison of melanin yield, color value and pullulan yield before and after adaptive evolution.
