# Supplementary material for: Investigation into the Production of Melanin from By-Products of Huangjiu Brewing
Source: Foods. 2024 Sep 26;13(19):3063. doi: 10.3390/foods13193063 (PMC11475479; doi:10.3390/foods13193063)
Supplement: Supplementary file 1 [file foods-13-03063-s001.zip › foods-3206366-supplementary.pdf]

foods

Supplementary

Table S1. ITS universal primer sequence

Primer	Sequences (5′→3′)
ITS1	TCCGTAGGTGAACCTGCGG
ITS4	GCTGCGTTCTTCATCGATGC

Table S2. Primers used in this study

Gene ID	Name	Sequence (5′ to 3′)
FKS	FKS-F	AGAAGACCGAGAAGGACACTGC
	FKS-R	TCTGAGAACGGAGCGATGCC
PKS	PKS-F	AAGCCTGAGGAGAAGCTCCAC
	PKS-R	CTGCTGGGGAATGTGGACATT
Cmr1	Cmr1-F	GGTCACTCGTTCACCAGGGA
	Cmr1-R	TGGAATTAGCCCGAGTTCTGAA

Table S3. Comparison of melanin yield, color value and pullulan yield before and after adaptive evolution

Generation	Melanin yield (g/L)	Color value	Pullulan yield (g/L)
0	5.91±0.21	108±2	5.06±0.35
1	5.96±0.18	108±4	5.09±0.26
2	6.05±0.22	108±2	5.18±0.23
3	6.23±0.26	109±3	5.36±0.12
4	6.59±0.18	109±2	5.62±0.24
5	6.89±0.24	110±2	6.02±0.21
6	7.02±0.19	110±1	6.65±0.20
7	7.94±0.19	111±2	7.22±0.21
8	8.72±0.25	112±3	7.22±0.18
9	8.07±0.31	110±1	7.20±0.14
10	8.06±0.26	110±3	7.15±0.22
